# Three-year follow-up of a self-administered Australian pelvic floor questionnaire validated in Chinese pregnant and postpartum women

**DOI:** 10.1007/s00192-022-05077-w

**Published:** 2022-01-17

**Authors:** Yuqing Hou, Baoqin Tong

**Affiliations:** Gynecology Department, Women’s Hospital School of Medicine Zhejiang University, No.1 Xueshi RoadZhejiang Province, Hangzhou, 310006 China

**Keywords:** Chinese, Pelvic floor disorders, Questionnaire, Validation studies, Follow-up

## Abstract

**Introduction and hypothesis:**

The aim of this study was to verify whether the Chinese version of the self-administered Australian Pelvic Floor Questionnaire (APFQ) can assess the changes in symptoms over a long time period (responsiveness) and to verify the reliability and validity of the questionnaire after 3 years of follow-up.

**Methods:**

The questionnaire was completed by 146 women using the WeChat platform after 3 years of follow-up. Reliability was assessed through internal consistency (Cronbach’s alpha). Construct validity was evaluated by significantly distinguished differences in symptom scores between women who did and did not subjectively suffer bothersome symptoms. Responsiveness was evaluated in 146 women. The effect size (ES) and standardized response mean (SRM) were used to assess the degree of responsiveness.

**Results:**

Of the 146 women, all completed the questionnaire through the WeChat platform without missing any items. Reliability, Cronbach’s alpha for the four domains and total APFQ were: bladder function 0.78, bowel function 0.71, pelvic organ prolapse 0.78, sexual function 0.68 and total APFQ 0.84. Concerning construct validity, the APFQ significantly distinguished the symptom scores between women who did and did not subjectively suffer from bothersome symptoms, and the score difference was 1.1–1.6 points, 1.2 points, 2.0–3.7 points and 1.4 points, respectively. For responsiveness, three domains showed slight (bowel domain) to moderate (bladder domain, sex domain) sensitivity to change. ES and SRM ranged from 0.21 to 0.75 and 0.16 to 0.60, respectively.

**Conclusions:**

The Chinese version of the self-administered APFQ is reliable and valid and can monitor the changes in symptoms over time.

## Introduction

Pelvic floor disorders (PFDs) refer to a broad range of clinical symptoms caused by changes in the anatomical structure of the pelvic floor muscles. They include urinary incontinence (UI), anal incontinence (AI), pelvic organ prolapse (POP) and sexual dysfunction (SD) [1]. These changes might be related to age, obesity, menopause, pregnancy and childbearing [2]. As the clinical symptoms of pelvic floor dysfunction diseases are diverse and involve a wide range of disciplines, it is difficult to determine the overall incidence of multiple diseases [3]. A longitudinal study of PFDs after childbirth showed that the rates of cases of PFDs after 9 years of follow-up were 11.4% for stress urinary incontinence (SUI), 13.8% for AI and 12.8% for POP [4]. Greater discomfort due to PFD symptoms is correlated with a worse quality of life (QoL) [5]. Perinatal women have limited knowledge of PFD, and we need to identify high-risk women to develop prevention strategies [6].

Although a comprehensive medical history and physical examination are important parts of a comprehensive evaluation, the proper use of diagnostic tools helps to perform a correct and complete analysis of patients with PFD. Questionnaires as well as diagnostic and physiologic tests can assess the severity of symptom distress and impact on QoL [7].

Patient-reported outcome (PRO) is a term used to describe a patient’s perception of their health, QoL or function related to a specific disease process or intervention. As healthcare increasingly transforms to a holistic patient-centered approach, PROs are the mainstay of the evaluation of women with PFD. Patient-reported outcome measures (PROMs) provide clinicians and researchers with a sufficient amount of information to enable clinicians to evaluate the patient’s symptoms and QoL in an objective way, while providing researchers with objective research tools that are easy to analyze. In the field of female PFD, there are many PROMs to assess different aspects of the diseases. We should choose the appropriate PROMs according to our research purposes [8].

A self-administered symptom and condition-specific quality of life questionnaire was introduced to obtain a patient-centered view of pelvic floor symptoms and their severity. The self-administered Australian Pelvic Floor Questionnaire (APFQ) comprehensively integrates four domains to assess bladder, bowel, prolapse and sexual symptoms, their severity, bother and impact on quality of life [9]. It has recently been validated in pregnant and postpartum women and designed to facilitate clinical assessment of pelvic floor symptoms. The questionnaire was initially in English and has been translated into German, Serbian, French, Turkish and Chinese [10–14].

Our research group previously evaluated the psychometric properties (including reliability, validity and responsiveness) of the self-administered Australian Pelvic Floor Questionnaire in Chinese pregnant and postpartum women during the third trimester of pregnancy, 2 month and 6 month follow-up. During the third trimester of pregnancy and 2 month follow-up, for construct validity, there was statistically significant difference in the symptom scores of women with and without subjective suffering of bothersome symptoms involving bladder function, bowel function, prolapse and sexual function. The total Cronbach’s alpha coefficients of the questionnaire in pregnancy and postpartum periods were 0.8, 0.9 and 0.9, respectively. Regarding responsiveness, the Chinese version of APFQ can track changes in the bladder function domain for women with standardized response mean equal to 0.6 during the third trimester of pregnancy to 2 months follow-up and also detect changes in bowel function domain with standardized response mean equal to 0.2 during 2–6 months of follow-up. The Chinese version of the self-administered APFQ had satisfactory reliability and validity and can longitudinally monitor changes in pelvic floor symptoms during pregnancy and postpartum periods.

The aim of the present study was to verify whether the Chinese version of the self-administered APFQ can evaluate changes in symptoms over time (responsiveness). At the same time, we also verified the reliability and validity of the questionnaire after 3 years of follow-up.

## Materials and methods

The Chinese version of the self-administered Australian pelvic floor questionnaire has recently been validated in pregnant and postpartum women. It is composed of 42 questions, divided into 4 independent domains: bladder function, bowel function, prolapse symptoms and sexual function. The higher the score is, the more severe the pelvic floor symptoms. The cross-cultural adaptation of APFQ, recruitment of participants and psychometric characteristics of APFQ in the third trimester of pregnancy, 2 months and 6 months after childbirth have been previously reported [14]. A total of 316 women in the third trimester of pregnancy participated in the original study. The mean age was 29.7 (SD 3.4) years; 306 (96.8%) and 274 (86.7%) women completed the questionnaire using the WeChat platform 2 and 6 months after childbirth, respectively.

All 316 participants of the original study received a message with a link to a questionnaire. The questionnaire, sent through the WeChat platform, was sent approximately 3 years after participants had been included in the initial study. In the present study, we analyzed all of the women who had completed the questionnaire.

All participants provided their oral informed consent. All women included did not receive any intervention associated with their participation. This research project was reviewed and approved by the Research Ethics Committee of the hospital involved. Collected data were well protected. Questionnaires used in samples were strictly anonymous.

Reliability refers to the degree of consistency or accuracy of the results obtained using a certain research tool. To assess the reliability, we calculated internal consistency of the defined domains. Cronbach’s alpha coefficient describes the homogeneity or intrinsic correlation between items in each of the four domains of the APFQ. A value ≥ 0.7 is generally considered to describe a high degree of internal consistency of the items [15].

Validity refers to the degree to which a research tool can truly reflect the concept it expects to study. We use statistical analysis to ensure that the APFQ can significantly distinguish women with or without subjectively bothersome pelvic floor symptoms in the third trimester of pregnancy and 3 years after childbirth, thereby verifying the construct validity of the questionnaire. The domain score for women with and without subjective suffering was compared and corroborated by the minimal important difference (MID). The MID of the APFQ ranged from 1.0 to 1.3 in the domains in the previous studies [16].

Responsiveness is defined as the ability of a questionnaire to detect clinically important changes over time, even if these changes are small [17]. We consider responsiveness to be a measure of longitudinal validity. The sensitivity of the questionnaire was analyzed by trying to detect changes in APFQ scores in the third trimester of pregnancy, 2 months, 6 months and 3 years after childbirth.

Distribution-based methods include paired *t*-test comparisons and calculation of the effect size [mean raw score change/standard deviation of baseline score and standardized response mean (mean raw score change/standard deviation of change)]. Effect size (ES) and standardized response mean (SRM) were used to demonstrate the degree of responsiveness [18]. Effect sizes and standardized response means of 0.2–0.49 are regarded as small, 0.5–0.8 as medium and > 0.8 as large [19].

## Statistical analysis

Statistical Package for Social Science version 20.0 was applied in this study. Descriptive data are expressed as mean (standard deviation, SD), median (range) and frequencies (percentages). The Mann-Whitney U test was used for comparisons of independent samples. A paired *t*-test and Wilcoxon signed-rank test were used for comparisons of paired samples. The significance level was set at *P* < 0.05.

## Results

**Fig. 1:**
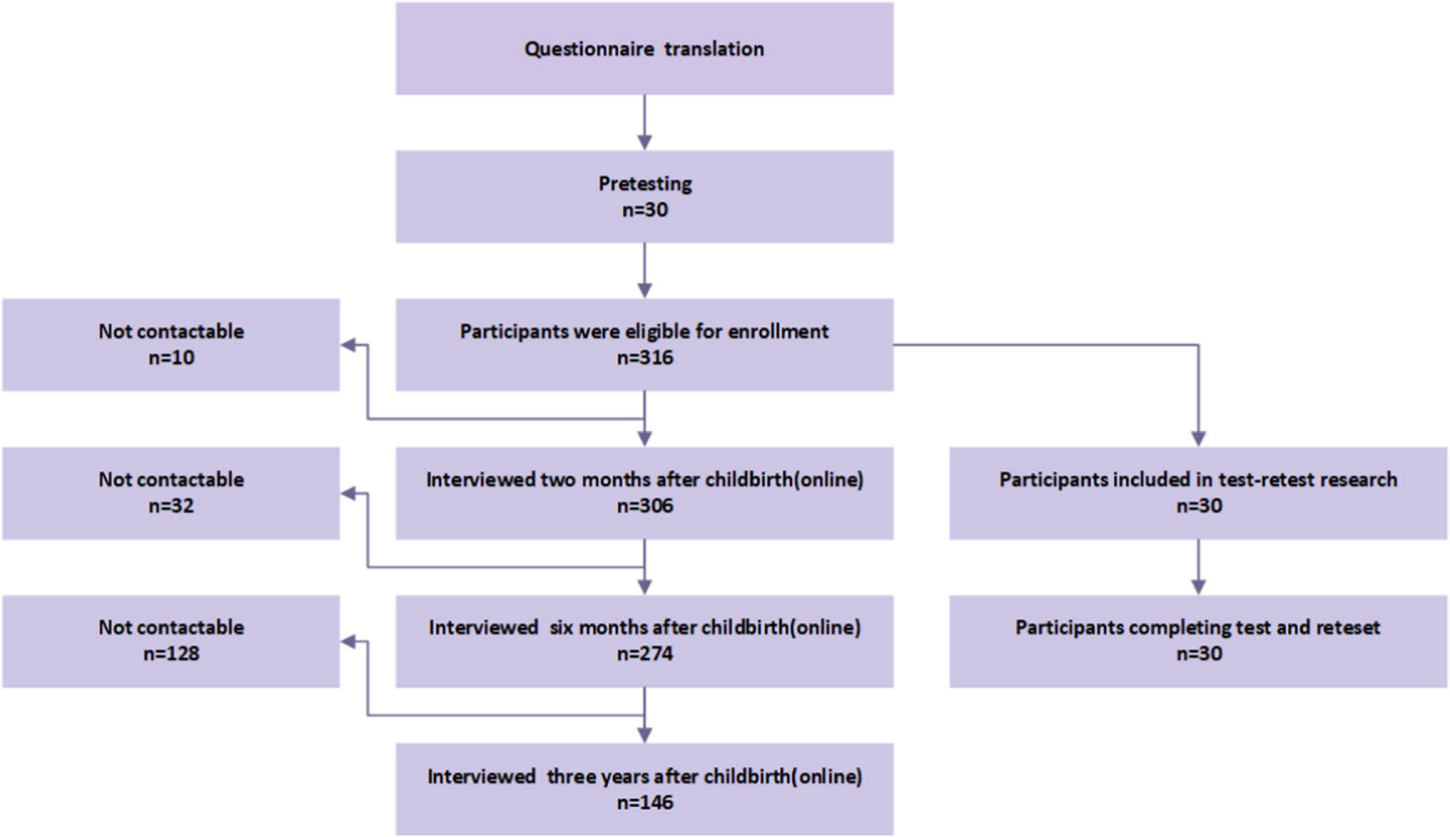
Study process of the Australian Pelvic Floor Questionnaire (APFQ)

Of the 316 women who participated in the third trimester of pregnancy in the original study, 146 women responded at 3 years. Thus, loss to follow-up was 53.8% (170/316). Of the 146 women, all completed the questionnaire through the WeChat platform without missing any items. Figure 1 shows the study process.

**Fig. 2:**
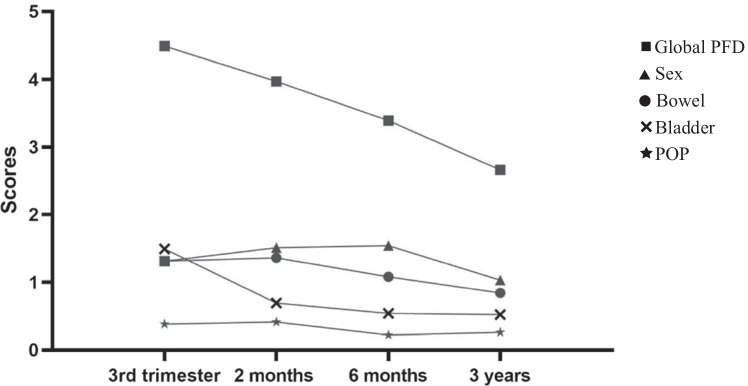
Changes in the mean values for total APFQ, bladder, bowel, POP and sex domains during the third trimester, 2 months, 6 months and 3 years after childbirth.

The changes in the scores of each domain and the total APFQ scores in different time periods are shown in Fig. 2. The total scores of the APFQ were the highest in the third trimester of pregnancy, and it showed a downward trend at 2 months, 6 months and 3 years after childbirth. The bladder function scores at 2 months after childbirth showed a downward trend compared with the scores in the third trimester of pregnancy. The scores at 6 months after childbirth were slightly lower than those at 2 months after childbirth, and the scores at 3 years after childbirth were the lowest. The scores of bowel function domain increased slightly at 2 months after childbirth and decreased at 6 months and 3 years after childbirth. The scores of the prolapse symptom domain increased slightly 2 months after childbirth and showed a downward trend at 6 months after childbirth, followed by a slight increase at 3 years after childbirth. The scores of the sexual function domain increased in the 2 months and 6 months after childbirth, and the scores decreased in the 3 years after childbirth. Since the scores of the sexual function domain and bowel function domain were both 1.31 (mean values) in the third trimester of pregnancy, the two starting points coincide in Fig. 2.

### Internal consistency

During the third trimester of pregnancy, Cronbach alpha values for the four domains were: bladder function 0.75, bowel function 0.73, pelvic organ prolapse 0.63 and sexual function 0.71. The total questionnaire score was 0.83.

Two months after childbirth, Cronbach alpha values for the four domains of the APFQ were: bladder function 0.82, bowel function 0.78, pelvic organ prolapse 0.62 and sexual function 0.76. The total APFQ was 0.89.

Six months after childbirth, Cronbach alpha values for the four domains were: bladder function 0.78, bowel function 0.74, pelvic organ prolapse 0.66 and sexual function 0.72. The total questionnaire score was 0.87.

Finally, 3 years after childbirth, the Cronbach alpha values in the four domains were: bladder function 0.78, bowel function 0.71, pelvic organ prolapse 0.78 and sexual function 0.68. The total questionnaire score was 0.84.

In the the third trimester of pregnancy, 2 months and 6 months after childbirth, all of the Cronbach alpha values exceeded 0.7, except for the pelvic organ prolapse domain. However, 3 years after childbirth, the Cronbach alpha value of the pelvic organ prolapse domain was 0.78. At the same time, the Cronbach alpha coefficient of the sexual function domain was 0.68, which was the lowest value among all time points (Table 1).

**Table 1:** Internal consistency: scores and Cronbach α values of the APFQ during the third trimester of pregnancy, 2 months, 6 months and 3 years after childbirth

Domains	Time	*N*	Score points	Cronbach’s α
Bladder	3rd trimester	316	1.11(0.00–6.00)	0.75
	2 months	306	0.44(0.00–5.56)	0.82
	6 months	274	0.33(0.00–3.78)	0.78
	3 years	146	0.44(0.00–3.78)	0.78
Bowel	3rd trimester	316	1.18(0.00–4.41)	0.73
	2 months	306	1.18(0.00–6.18)	0.78
	6 months	274	0.88(0.00–4.41)	0.74
	3 years	146	0.88(0.00–3.24)	0.71
Prolapse	3rd trimester	316	0.00(0.00–5.33)	0.64
	2 months	306	0.00(0.00–5.33)	0.62
	6 months	274	0.00(0.00–4.00)	0.66
	3 years	146	0.00(0.00–6.67)	0.78
Sex	3rd trimester	247	0.95(0.00–8.50)	0.71
	2 months	217	1.43(0.00–7.14)	0.76
	6 months	216	1.43(0.00–4.76)	0.72
	3 years	76	0.95(0.00–3.33)	0.68
Global PFD	3rd trimester	247	4.08(0.22–14.46)	0.83
	2 months	217	3.49(0.00–14.78)	0.89
	6 months	216	2.85(0.00–14.63)	0.87
	3 years	76	2.50(0.00–10.52)	0.84

PFD: pelvic floor dysfunction

The score points are presented as median (range)

If any of the domains lacked a sub-score, the total score was not calculated

In the sexual function domain (Table1), there were 247/316 samples in the third trimester of pregnancy. Sixty-nine of 316 women were not sexually active; among them, in 61 this was because of "other reasons," in 6 because "I’m not interested," in 1 because it was "too painful," in 1 because of "vaginal dryness" and in 1 because of "embarrassment due to prolapse or incontinence," so the sexual function domain score of the last patient was given 8.5 (18/21) points. There were 217/306 samples for the 2 months after childbirth; 89/306 respondents were not sexually active. Of these, in 61 this was for "other reasons," in 17 because "I’m not interested," in 2 because "I do not have a partner," in 4 because it was "too painful" and in 5 because of "vaginal dryness." The issues were not related to pelvic floor disorders, and the sexual function domain score was not calculated. There were 216/274 samples for the 6 months after childbirth; 58/274 respondents were not sexually active. Of these, in 21 this was because of "other reasons," in 19 because "I’m not interested," in 8 because "I do not have a partner," in 5 because it was "too painful" and in 5 because of "vaginal dryness." The issues were not related to pelvic floor disorders, and the sexual function domain score was not calculated. There were 76/146 samples for the 3 years after childbirth; 70/146 respondents were not sexually active. Of these, in 29 this was for "other reasons," in 27 because "I’m not interested," in 7 because "I do not have a partner," in 2 because it was "too painful" and in 3 because of "vaginal dryness." The issues were not related to pelvic floor disorders, and the sexual function domain score was not calculated.

### Construct validity

The APFQ significantly distinguished the symptom scores between who did and did not subjectively suffer from bothersome symptoms (*P* < 0.01, Table 2). The score difference was 1.1–1.6 points, 1.2 points, 2.0–3.7 points and 1.4 points, respectively, consistent with the minimal important difference (MID) of each domain of the APFQ reported by Baessler et al. [16].

**Table 2:** Construct validity: score difference between women who did and did not subjectively suffer from bothersome symptoms during the third trimester of pregnancy and 3 years after childbirth

Domains	Time	Bother	*N*	Score points	P^*^ value
Bladder	3rd trimester	No	238	1.1 (0.0–3.8)	< 0.01
		Yes	78	2.2 (0.9–6.0)	
	3 years	No	129	0.2 (0.0–2.4)	< 0.01
		Yes	17	1.8 (0.7–3.8)	
Bowel	3rd trimester	No	169	0.6 (0.0–2.4)	< 0.01
		Yes	147	1.8 (0.9–4.4)	
	3 years	No	112	0.3 (0.0–2.1)	< 0.01
		Yes	34	1.5 (0.6–3.2)	
Prolapse	3rd trimester	No	274	0.0 (0.0–2.0)	< 0.01
		Yes	42	2.0 (1.3–5.3)	
	3 years	No	140	0.0 (0.0–2.7)	< 0.01
		Yes	6	3.7 (2.0–6.7)	
Sex	3rd trimester	No	24	1.0(0.0–2.4)	< 0.01
		Yes	75	2.4(0.5–4.3)	
	3 years	No	70	1.0 (0.0–3.3)	< 0.01
		Yes	6	2.4 (1.9–3.3)	

The score points are presented as median (range)

^*^P, Mann-Whitney U test

### Responsiveness

Three domains showed slight (bowel domain) to moderate (bladder domain, sex domain) sensitivity to change (Table 3). In the third trimester of pregnancy and 2 months after childbirth, in the domain of bladder function, ES = 0.75, SMR = 0.60, *P* < 0.001. In the 2 months and 6 months after childbirth, in the bowel function domain, ES = 0.29, SMR = 0.22, *P* < 0.001, and in the 6 months and 3 years after childbirth, ES = 0.26, SMR = 0.24, *P* < 0.05. In the 6 months and 3 years after childbirth, in the sexual function domain, ES = 0.45, SMR = 0.44, *P* < 0.05, and in the third trimester of pregnancy and 2 months after childbirth, ES = 0.21, SMR = 0.16, *P* < 0.05. In the prolapse symptoms domain, only in the 2 months and 6 months after childbirth, ES = 0.19, SMR = 0.14, *P* < 0.05. Prolapse symptoms did not change significantly over time.

**Table 3:** Responsiveness: effect size and standardized response mean of the APFQ showed changes at different time points

Domain	T1 score	T2 score	Mean score	Effect size change	Standardized response mean	*P* ^*^
Bladder^a^	1.46 ± 1.02	0.68 ± 0.88	-0.77 ± 1.28	0.75	0.60	< 0.001
Bladder^b^	0.68 ± 0.88	0.57 ± 0.72	-0.13 ± 1.12	0.15	0.12	0.074
Bladder^c^	0.57 ± 0.72	0.62 ± 075	-0.01 ± 0.72	0.01	0.01	0.965
Bowel	1.28 ± 0.89	1.36 ± 1.04	0.10 ± 1.36	0.11	0.07	0.213
Bowel	1.36 ± 1.04	1.05 ± 0.84	-0.30 ± 1.34	0.29	0.22	< 0.001
Bowel	1.05 ± 0.84	0.86 ± 0.78	-0.22 ± 0.90	0.26	0.24	< 0.05
Prolapse	0.38 ± 0.79	0.41 ± 0.89	0.03 ± 1.15	0.04	0.03	0.452
Prolapse	0.41 ± 0.89	0.26 ± 0.66	-0.17 ± 1.19	0.19	0.14	< 0.05
Prolapse	0.26 ± 0.66	0.33 ± 0.91	0.02 ± 0.84	0.03	0.02	0.842
Sex	1.31 ± 1.15	1.51 ± 1.30	0.24 ± 1.54	0.21	0.16	< 0.05
Sex	1.51 ± 1.30	1.54 ± 1.16	0.08 ± 1.64	0.06	0.05	0.525
Sex	1.54 ± 1.16	1.03 ± 0.97	-0.52 ± 1.17	0.45	0.44	< 0.05

The scores are presented as mean (standard deviation)

Bladder^a^ represents the bladder function domain score from in the third trimester of pregnancy to 2 months after childbirth

Bladder^b^ represents the bladder function domain score from 2 months to 6 months after childbirth

Bladder^c^ represents the bladder function domain score from 6 months to 3 years after childbirth

Bowel, prolapse and sex domains were the same as above

T = time points

P^*^, paired *t*-test and Wilcoxon signed-rank test

## Discussion

We have validated the self-administered APFQ in the Chinese pregnant and postpartum women during the third trimester of pregnancy and 2 months and 6 months after childbirth. The questionnaire had satisfactory reliability and validity and can longitudinally monitor changes in pelvic floor symptoms during the pregnancy and postpartum periods. Three years after childbirth, we once again conducted a follow-up survey of the initial participants and distributed questionnaires through the WeChat platform.

Many scientific data indicate that pregnancy and childbirth are the main risk factors for female pelvic floor disorders (urinary incontinence, anal incontinence, pelvic organ prolapse, dyspareunia) [20]. During pregnancy many women experience frequent urination symptoms, which are caused by an enlarged uterus compressing the bladder. Data from the present study provided a more complete picture of four domain scores and total PFD scores over time and illustrated differences in the progression of the four pelvic floor disorders over time. The bladder domain score and total APFQ score showed a downward trend compared with baseline. The scores of the bowel, POP and sex domains were ultimately lower than the baseline scores. However, at 2 and 6 months postpartum, the scores of some of the above three domains increased slightly, and the scores of some domains decreased slightly. The sequelae of obstetric perineal and anal sphincter tears may last for some time and have long-term effects. Women who have been diagnosised with short-term bowel, bladder and healing problems should actively seek help from healthcare providers at 4 to 6 weeks. The multidisciplinary team can provide a structured means to enable pregnant and postpartum women with pelvic floor symptoms to receive specialized counseling and treatment, thereby reducing the incidence of PFD symptoms in postpartum women [21].

Regarding reliability, we used Cronbach’s α coefficient to evaluate the internal consistency of the APFQ. The total Cronbach’s α coefficient of the APFQ during the third trimester of pregnancy, 2 months, 6 months and 3 years after childbirth was > 0.8, indicating that the questionnaire items had good correlation and internal consistency. However, the Cronbach’s α coefficients for the prolapse symptom domain measured in the first three measurements were 0.64, 0.62 and 0.66, respectively. This may be because the number of items in the prolapse symptom domain is only 5. When the number of items is less than ten, the Cronbach’s alpha value may be low [22]. However, in the last measurement, the Cronbach’s α coefficient of the prolapse symptom domain was 0.78. We still need more research to verify the reliability of this domain and make it more suitable for the Chinese population in the future.

Regarding construct validity, we used the significant differences between domain symptom scores for women with and without bothersome symptoms for confirmation. The minimal important difference (MID) of a questionnaire indicates the change in symptoms that makes a meaningful difference to the patient. MID helps explain changes in PFD symptoms over time. During the third trimester of pregnancy and 3 years after childbirth, the symptom scores of bladder function, bowel function, prolapse symptoms and sexual function domains were significantly higher than for the women who reported "not at all," and the score difference was 1.1–1.6 points, 1.2 points, 2.0–3.7 points and 1.4 points, respectively. The above minimal important difference values are all > 1, corresponding to the minimal important differences (MID) for each domain reported by Baessler et al. and Hou et al. [14, 16], and these can be considered clinically important differences. In future studies, we can detect whether a woman needs treatment by assessing bothersomeness rather than treating her after reaching a certain symptom score.

There have been no other validated questionnaires in the Chinese language covering all areas of pelvic floor disorders, so we did not calculate the correlation between the APFQ and other reference measurements to estimate criterion validity. In the future, it will be necessary to find the "gold standard" questionnaire in the field of pelvic floor disorder treatment to verify the correlation between the APFQ and existing standards.

Responsiveness means that when the internal and external environment changes, the measurement outcomes can react sensitively. It is used to evaluate changes in symptoms over time and treatment effects. In this study, the bladder function and sex domains were moderately responsive and could clearly identify changes in PFD symptoms in the third trimester of pregnancy and 2 months after childbirth, as well as the changes in PFD symptoms within 6 months and 3 years after childbirth, consistent with the findings for the French version of the APFQ [12]. The bowel function domain had low reactivity and could detect changes of symptoms in women from 2 months to 3 years after childbirth. Prolapse symptoms were stable over time with a standardized mean response < 0.2 between successive assessments.

The Chinese version of the self-administered APFQ fully integrated the four domains of bladder function, bowel function, prolapse symptoms and sexual function, which had satisfactory reliability, validity and responsiveness in the third trimester of pregnancy, 2 months, 6 months and 3 years after childbirth.

The limitations of this study include the psychometric properties of reliability in prolapse symptom domains, which still need further validation. Second, we need to calculate the correlation between the APFQ and the existing gold standard to verify the criterion validity.

## Conclusions

The Chinese version of the self-administered APFQ has satisfactory reliability and validity and can be used as a longitudinal monitoring tool for pelvic floor symptoms in women.
